# Supplementary material for: Ubiquitous strong electron–phonon coupling at the interface of FeSe/SrTiO3
Source: Nat Commun. 2017 Feb 10;8:14468. doi: 10.1038/ncomms14468 (PMC5311057; doi:10.1038/ncomms14468)
Supplement: Supplementary Information — Supplementary Figures 1-6 [file ncomms14468-s1.pdf]

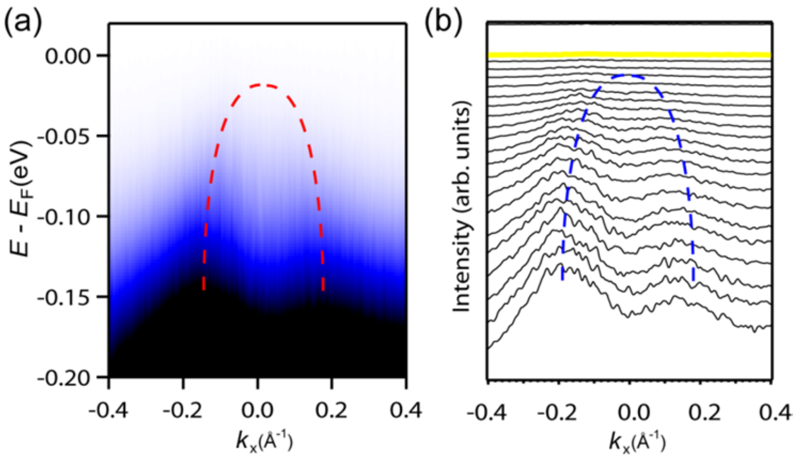

Supplementary Figure 1

The bands at the Gamma point.

(a) Energy-momentum intensity map of the Gamma point. Dashed line shows the position of the hole band. **(b)** The momentum distribution curves of (**a**). The yellow line shows the E_F level, and the blue line shows the position of the band. By fitting the momentum distribution curves (MDCs), we estimate the top of hole band is located at ~20 meV below Fermi level.

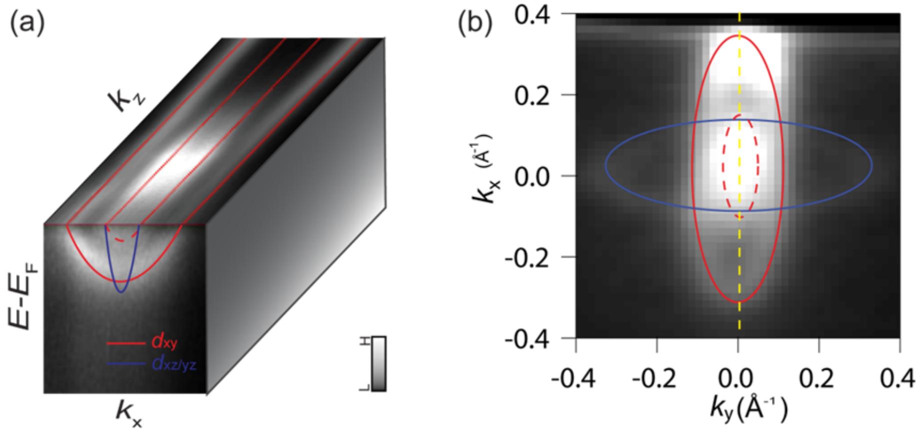

Supplementary Figure 2

The surface electronic structure of STO(110).

(a) Energy-momentum intensity map of STO(110), the d_{xy} and $d_{yz/xz}$ like bands are marked by the red and blue curves respectively. Photon energy dependence shows no dispersion of along k_z as shown on the top face. The three dimensional k_x-k_z-E (k_y=0) APRES data of STO(110) with carrier density of 6.0×10^{13} cm^{-2} (estimated from the Luttinger's volume). The carrier density associated with data shown in this figure is relatively high but has not reached the saturation level – the doping level beyond which carriers begin to spread into the bulk. The solid and dashed red parabolic curves plotted on the front face are both d_{xy}-like bands. The one at higher binding energy is the main band, while that at lower binding energy is the quantum well subband. The blue curve on the front face marks a $d_{xz}/_{yz}$-like band which has comparatively weak intensity due to unfavorable ARPES matrix elements. We measure the k_z dependence by varying the photon energies from 45eV to 75eV. The resulting k_z dependence of the Fermi surface is shown on the top face. Clearly the Fermi momenta show no measurable k_z dependence indicating the 2D nature of the observed bands. **(b)** The Fermi surface map of STO(110) shows two cigar-shaped pockets crossing each other. The solid curves show the Fermi surface of the main d_{xy} and $d_{xz/yz}$ bands in the k_x-k_y plane at photon energy of 48eV. Two perpendicular cigar-shaped Fermi surfaces are observed. In the same figure the dashed red curve marks the Fermi surface of the d_{xy} quantum well subband.

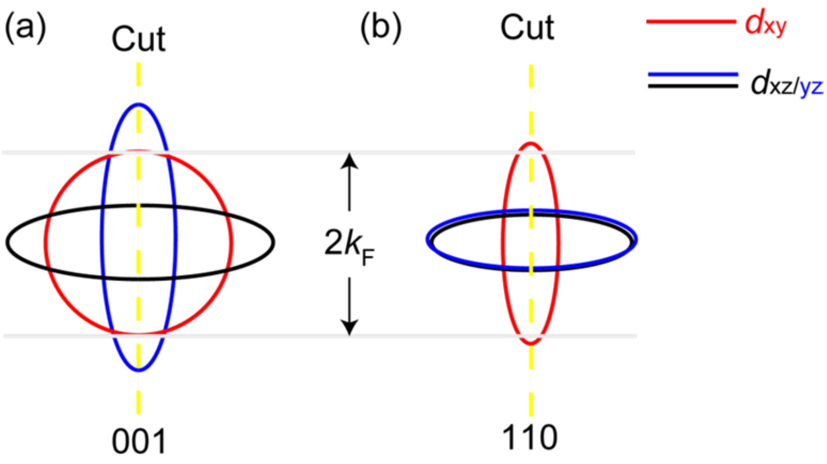

Supplementary Figure 3

The Schematic of Fermi surfaces at STO(001) and STO(110) with similar k_F.

(**a**) The Fermi surface of STO(001) consists of circular d_{xy} orbital and two anisotropic $d_{xz/yz}$ orbitals that crossing with each other. (**b**) The Fermi surface of STO(110) shows three similar elliptical pockets of d_{xy} and $d_{xz/yz}$. In the schematic we choose similar k_F of d_{xy} orbital for comparison. The area covered by all the three orbitals in STO(001) is more than two times than that of STO(110).

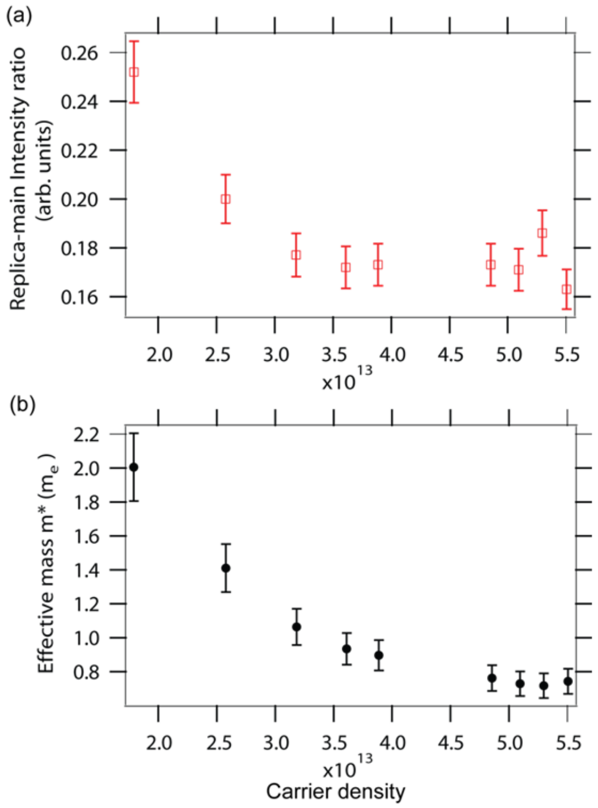

Supplementary Figure 4

The intensity ratio between the replica and the main band and the effective mass as a function of carrier density.

(a) The evolution of replica intensities versus the carrier density. (**b)** The evolution of effective mass of the main band versus the carrier density. We estimate the effective mass from the parabolic d_{xy} bands at different carrier densities. The result is $2m_e$ at lower carrier density which amounts to an effective mass enhancement m^*/m_0 ~3.3. At higher doping density the effective mass is 0.8 m_e which gives m^*/m_0 ~ 1.3. If one attributes the change in the effective mass to the change in the electron-phonon coupling strength this is consistent with the trend deduced from (**a)**. The error bars in the figures are estimated from both the energy resolution and the fitting error. The counts of band intensity is in the scale of 10^3.

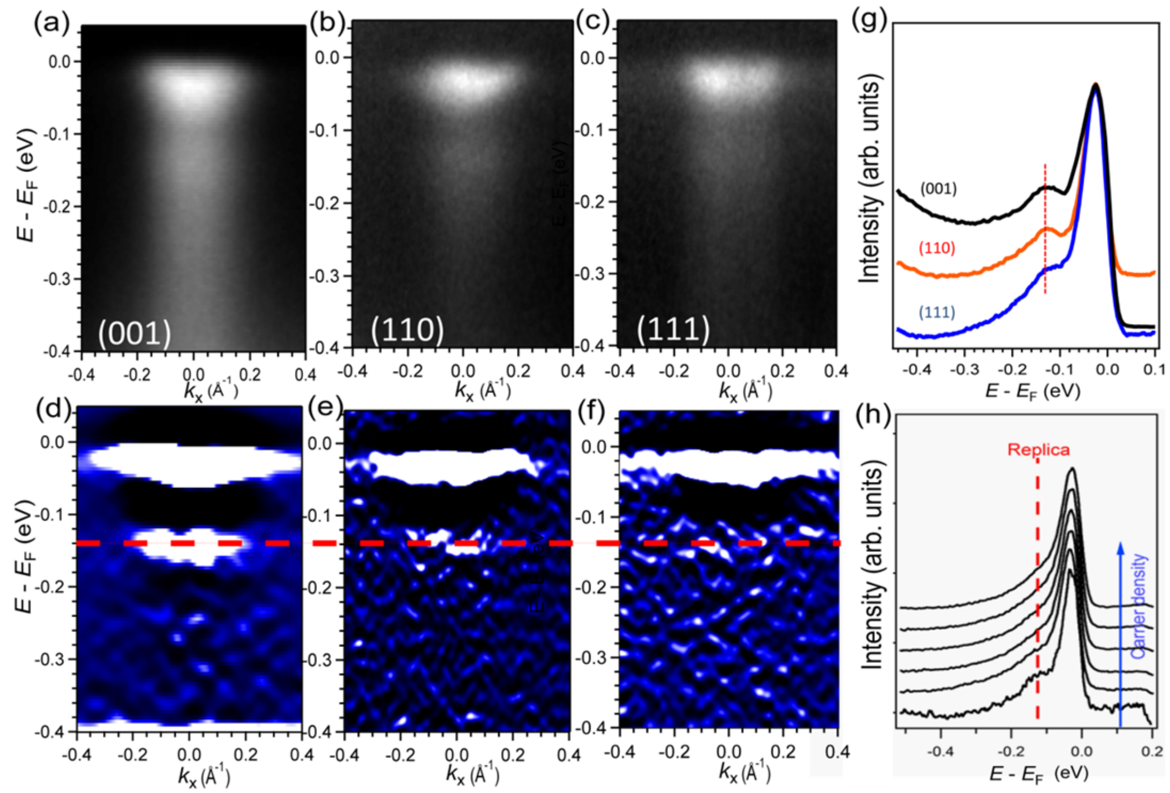

Supplementary Figure 5

The comparative study of STO surface (001), (110) and (111).

(**a**)-(**c**). The raw data of the 2DEG band and the replica band at higher binding energy. (**d**)-(**f**) shows the 2nd energy derivative of corresponding upper images. We can observe the replica bands of all the three surfaces, while the data quality of 110 and 111 are poorer than 001. In the energy distribution curves (EDCs) shown at (**g**), we can see the separation between replica bands and main bands are approximately same for all the three surfaces. (**h**) The EDCs at k_F as function of doping are presented. When the photo doping move towards higher electron density, the strength of electron-phonon coupling decreases. The ratio of replica band intensity to that of main band decreases as the doping level increases. The trend is similar to what we present in the main text for STO(110) surface.

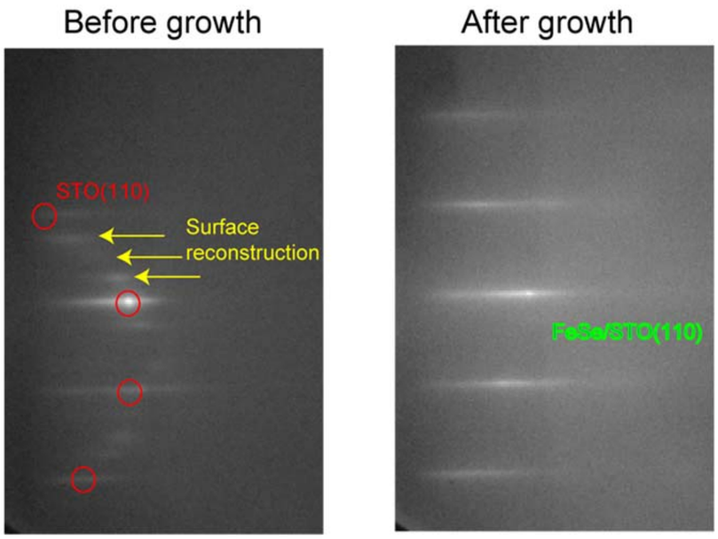

Supplementary Figure 6

The RHEED pattern before and after growth.

Here we show the reflection high-energy electron diffraction (RHEED) patterns before and after growth. After degassing at 450°C for 1hour, we gradually increase the temperature to 830°C, at which a new RHEED pattern appears. We suggest this is due to the oxygen vacancies formed with the surface (4×1) reconstruction. After the growth of monolayer FeSe on top, we saw the conventional thin and straight pattern that indicates a good quality film.
